# Combined genomic-proteomic approach in the identification of *Campylobacter coli* amoxicillin-clavulanic acid resistance mechanism in clinical isolates

**DOI:** 10.3389/fmicb.2023.1285236

**Published:** 2023-11-09

**Authors:** Francis Deforet, Quentin Jehanne, Lucie Bénéjat, Johanna Aptel, Roxane Prat, Chloé Desbiolles, Astrid Ducournau, Marine Jauvain, Richard Bonnet, François Vandenesch, Jérôme Lemoine, Philippe Lehours

**Affiliations:** ^1^Institut des Sciences Analytiques, Université Claude Bernard Lyon 1, Lyon, France; ^2^Bacteriology Department, CHU de Bordeaux, National Reference Center for Campylobacters and Helicobacters, Bordeaux, France; ^3^Institut des Agents Infectieux, Hospices Civils de Lyon, Lyon, France; ^4^Bordeaux Institute of Oncology, BRIC U1312, INSERM, Université de Bordeaux, Bordeaux, France; ^5^Laboratoire Associé CNR de la Résistance aux Antibiotiques, CHU de Clermont-Ferrand, Clermont-Ferrand, France

**Keywords:** AMR, amoxicillin-clavulanic acid, *Campylobacter*, beta-lactamase, gene expression

## Abstract

**Introduction:**

Aminopenicillins resistance among *Campylobacter jejuni* and *Campylobacter coli* strains is associated with a single mutation in the promoting region of a chromosomal beta-lactamase *bla_OXA61_*, allowing its expression. Clavulanic acid is used to restore aminopenicillins activity in case of *bla_OXA61_* expression and has also an inherent antimicrobial activity over *Campylobacter* spp. Resistance to amoxicillin-clavulanic acid is therefore extremely rare among these species: only 0.1% of all *Campylobacter* spp. analyzed in the French National Reference Center these last years (2017–2022).

**Material and methods:**

Whole genome sequencing with bioinformatic resistance identification combined with mass spectrometry (MS) was used to identify amoxicillin-acid clavulanic resistance mechanism in Campylobacters.

**Results:**

A G57T mutation in *bla_OXA61_* promoting region was identified in all *C. jejuni* and *C. coli* ampicillin resistant isolates and no mutation in ampicillin susceptible isolates. Interestingly, three *C. coli* resistant to both ampicillin and amoxicillin-clavulanic acid displayed a supplemental deletion in the promoting region of *bla_OXA61_* beta-lactamase, at position A69. Using MS, a significant difference in the expression of Bla_OXA61_ was observed between these three isolates and amoxicillin-clavulanic acid susceptible *C. coli*.

**Conclusion:**

A combined genomics/proteomics approach allowed here to identify a rare putative resistance mechanism associated with amoxicillin-clavulanic acid resistance for *C. coli.*

## Introduction

*Campylobacter jejuni* and *Campylobacter coli* are the most common cause of bacterial gastro-enteritis worldwide, before *Salmonella* (Chlebicz and Śliżewska, 2018). They are foodborne pathogens transmitted via the consumption of contaminated products, especially meat (mainly chicken, beef and pork). In Europe in 2021, campylobacteriosis accounted for more than 120,000 cases of illness (The European Union One Health, 2021) while in the USA, the number of *Campylobacter* infections is estimated at 1.5 million illnesses each year (Delahoy et al., 2023). Campylobacteriosis can cause symptoms such as abdominal pains, fever and diarrhea, which are significant risks of complications at the extreme ages of life (Fernández-Cruz et al., 2010). Antimicrobial therapy is considered in case of serious infections, but resistance to commonly used antimicrobials is of concern.

In 2022, 6,772 *C. jejuni* and 1,138 *C. coli* clinical isolates were tested at the French National Reference Center for Campylobacters and Helicobacters (Lehours et al., 2023) (NRCCH) showing 63.1 and 64% resistance to ciprofloxacin, 46.9 and 79.4% resistance to tetracycline as well as 33.5 and 29.1% resistance for ampicillin, respectively. However, resistance to macrolides and aminoglycosides remained very low, even though *C. coli* showed a concerning increase in erythromycin resistance through the emergence of new mechanisms (Jehanne et al., 2021). In France, 0.3% of *C. jejuni* and 7% of *C. coli* isolates are resistant to erythromycin and 0.4% of *C. jejuni* and 2.4% of *C. coli* isolates are resistant to gentamicin. In addition, less than 0.1% of both *C. jejuni* and *C. coli* are resistant to amoxicillin-clavulanic acid, a phenomena which is more common for *Campylobacter* closely related species, for instance *Aliarcobacter butzleri* (Zacharow et al., 2015).

Due to the excessive use of antimicrobials, especially in animal, specific genetic mechanisms of resistance have been selected among *C. jejuni* and *C. coli* (Griggs et al., 2005; Ladely et al., 2009; Qin et al., 2014; Florez-Cuadrado et al., 2017; Chen et al., 2018; Fabre et al., 2018; Elhadidy et al., 2019; Anampa et al., 2020; Greninger et al., 2020; Hormeño et al., 2020; Wallace et al., 2020; Jehanne et al., 2021). The G57T mutation in the promoting region of *bla_OXA61_* (Zeng et al., 2014) is, in particular, associated with ampicillin resistance. However, no amoxicillin-clavulanic acid resistance mechanism has yet been described. Amoxicillin-clavulanic acid can therefore be an antimicrobial of choice against campylobacteriosis, especially in case of bacteremia (Abay et al., 2014; Schiaffino et al., 2019; Tinévez et al., 2022). However, high levels of resistance can be found in some clinical isolates with minimum inhibitory concentration (MIC) reaching 256 mg/L or above. In the present study, 30 clinical isolates from the collection of the NRCCH were analyzed. Amoxicillin-clavulanic acid and ampicillin resistant and susceptible *C. jejuni* and *C. coli* were selected (i) to perform whole-genome sequencing (WGS), (ii) to quantify the expression of Bla_OXA61_ using mass spectrometry (MS) in order to (iii) identify the genetic mechanism associated with amoxicillin-clavulanic acid resistance. It allowed us to link Bla_OXA61_ expression levels to the sequence of its promoting region and especially amoxicillin-clavulanic acid resistance to the presence of a supplemental deletion at position A69 in *C. coli*.

## Materials and methods

### Isolates selection

A total of 30 isolates (12 *C. coli* and 18 *C. jejuni*), listed in Table 1 with their corresponding ENA accession number, were analyzed in the present study as well as two references (*C. jejuni* CCUG 11284 and *C. coli* CCUG 11283). The mean age and sex ratio (m/f) of the dataset were 38.4 years and 1.7, respectively. It is composed of clinical strains that have been isolated between 2017 and 2020 from stools and send to the French National Reference Center for Campylobacters and Helicobacters (NRCCH) (Bordeaux, France) by clinical laboratories participating to its surveillance network. Among the *C. jejuni* isolates, nine were ampicillin (AMP)-susceptible (S), and nine were AMP-resistant (R). They were all amoxicillin-clavulanic acid (AMC)-susceptible. Four of the 12 *C. coli* were AMP-S and eight were AMP-R. Three AMP-R *C. coli*, from 2017, 2018 and 2020, were resistant to amoxicillin-clavulanic acid (AMC). Species were identified by matrix-assisted laser desorption ionization-time of flight-mass spectrometry as already described (Bessède et al., 2011). Antimicrobial susceptibility to ampicillin, amoxicillin-clavulanic acid, ciprofloxacin, erythromycin, tetracycline, and gentamicin were performed by diffusion using EUCAST guidelines.^1^ Ampicillin and amoxicillin-clavulanic acid MICs were determined using Etest (bioMérieux, Marcy l’Etoile, France) and interpreted according to the cut-off values proposed by the CASFM: S ≤ 4 mg/L, R > 16 mg/L.^2^

**Table 1:** *C. coli* and *C. jejuni* French clinical isolates from 2017 to 2020 analyzed in the present study.

											Molecular resistance identification^f^			
Isolates	Species	ANI score^a^	Patient age	Patient sex	Source	Sample date	AMP MIC (mg/L)^b^	AMC MIC (mg/L)^c^	*blaOXA61* promoter^d^	ENA assembly accession^e^	CIP	ERY	TET	GEN
2017-1086H	*Campylobacter coli*	98.67	80	Female	Stools	Dec 2017	**>256 (R)**	**12 (R)**	G57T + ΔA69	GCA_958296275	GyrA D90N	23S A2075G	*tet(O)*	–
2018-0030H	*Campylobacter coli*	98.73	3	Male	Stools	Jan 2018	**>256 (R)**	**12 (R)**	G57T + ΔA69	GCA_958296255	GyrA D90N	23S A2075G	*tet(O)*	–
2020-0472	*Campylobacter coli*	98.71	69	Female	Stools	May 2020	**>256 (R)**	**256 (R)**	G57T + ΔA69	GCA_958295195	GyrA T86I	23S A2075G	*tet(O)*	–
2018-2008	*Campylobacter coli*	98.76	2	Male	Stools	Sep 2018	**128 (R)**	2 (S)	G57T	GCA_958296215	GyrA T86I	23S A2075G	*tet(O)*	–
2019-0242H	*Campylobacter coli*	99.06	16	Female	Stools	May 2019	**64 (R)**	1.5 (S)	G57T	GCA_958296265	GyrA T86I	23S A2075G	*tet(O-M-O)*	*APH(2″)-IIIa*
2019-0409	*Campylobacter coli*	98.72	34	Female	Stools	Mar 2019	**>256 (R)**	1.5 (S)	G57T	GCA_958296375	GyrA T86I	–	–	–
2020-0014H	*Campylobacter coli*	98.70	17	Male	Stools	Jan 2020	**>256 (R)**	3 (S)	G57T	GCA_958295415	GyrA T86I	–	*tet(O)*	–
2020-0548	*Campylobacter coli*	98.78	7	Female	Stools	May 2020	**96 (R)**	2 (S)	G57T	GCA_958296355	GyrA T86I	–	*tet(O)*	–
2018-1149	*Campylobacter coli*	99.02	43	Male	Stools	Jun 2018	4 (S)	1 (S)	*wt*	GCA_958296365	GyrA T86I	*erm(B)*	*tet(O)*	–
2019-2217	*Campylobacter coli*	98.92	1	Male	Stools	Sep 2019	2 (S)	1 (S)	*wt*	GCA_958295225	–	–	–	–
2020-0368	*Campylobacter coli*	98.76	89	Male	Stools	Apr 2020	4 (S)	1 (S)	*wt*	GCA_958295175	GyrA T86I	–	*tet(O)*	–
2020-0448H	*Campylobacter coli*	98.69	68	Female	Stools	Jul 2020	3 (S)	1 (S)	*wt*	GCA_958295215	–	23S A2074G	*tet(O)*	–
2018-0007	*Campylobacter jejuni*	97.67	29	Female	Stools	Jan 2018	**32 (R)**	<0.016 (S)	G57T	GCA_958295635	–	–	–	–
2018-0008	*Campylobacter jejuni*	97.68	8	Male	Stools	Jan 2018	**24 (R)**	<0.016 (S)	G57T	GCA_958295325	GyrA T86I	–	*tet(O)*	–
2018-0013	*Campylobacter jejuni*	97.53	52	Female	Stools	Jan 2018	**32 (R)**	<0.016 (S)	G57T	GCA_958295375	GyrA T86I	–	*tet(O)*	–
2018-0015	*Campylobacter jejuni*	97.38	48	Male	Stools	Jan 2018	**64 (R)**	<0.016 (S)	G57T	GCA_958295345	GyrA T86I	–	*tet(O)*	–
2018-0024H	*Campylobacter jejuni*	97.65	3	Male	Stools	Jan 2018	**64 (R)**	<0.016 (S)	G57T	GCA_958295235	GyrA T86I	–	*tet(O-32-O)*	–
2018-1793	*Campylobacter jejuni*	97.53	74	Female	Stools	Aug 2018	**32 (R)**	<0.016 (S)	G57T	GCA_958295365	GyrA T86I	23S A2075G	*tet(O-M-O)*	–
2019-0006H	*Campylobacter jejuni*	97.58	21	Male	Stools	Jan 2019	**64 (R)**	<0.016 (S)	G57T	GCA_958295285	GyrA T86I	–	*tet(O)*	–
2019-0008	*Campylobacter jejuni*	97.66	4	Male	Stools	Jan 2019	**24 (R)**	<0.016 (S)	G57T	GCA_958295355	GyrA T86I	–	–	–
2019-1193	*Campylobacter jejuni*	97.53	45	Male	Stools	Jun 2019	**32 (R)**	0.016 (S)	G57T	GCA_958295305	GyrA T86I	23S A2074C	*tet(O-32-O)*	–
2018-0009H	*Campylobacter jejuni*	97.63	66	Female	Stools	Jan 2018	3 (S)	<0.016 (S)	*wt*	GCA_958295385	GyrA T86I	–	–	–
2018-0014	*Campylobacter jejuni*	97.55	21	Male	Stools	Jan 2018	1.5 (S)	<0.016 (S)	*wt*	GCA_958295275	GyrA T86I	–	–	–
2018-0023H	*Campylobacter jejuni*	97.60	16	Male	Stools	Jan 2018	1.5 (S)	<0.016 (S)	*wt*	GCA_958295585	GyrA T86I	–	–	–
2018-0069H	*Campylobacter jejuni*	97.65	87	Male	Stools	Feb 2018	1 (S)	<0.016 (S)	*wt*	GCA_958295295	–	–	*tet(O)*	–
2018-0082H	*Campylobacter jejuni*	97.49	100	Male	Stools	Feb 2018	2 (S)	<0.016 (S)	*wt*	GCA_958295205	–	–	–	–
2018-0116	*Campylobacter jejuni*	97.72	67	Male	Stools	Jan 2018	2 (S)	<0.016 (S)	*wt*	GCA_958295245	GyrA T86I	23S A2075G	*tet(O)*	–
2019-0011	*Campylobacter jejuni*	97.68	22	Female	Stools	Jan 2019	2 (S)	<0.016 (S)	*wt*	GCA_958295255	GyrA T86I	–	*tet(O)*	–
2019-0026	*Campylobacter jejuni*	97.52	2	Male	Stools	Jan 2019	2 (S)	<0.016 (S)	*wt*	GCA_958295335	GyrA T86I	–	*tet(O)*	–
2019-0207	*Campylobacter jejuni*	97.14	58	Male	Stools	Feb 2019	2 (S)	<0.016 (S)	*wt*	GCA_958295265	GyrA T86I	23S A2074T	*tet(O)*	–

Genomes were assembled using SPAdes and species were confirmed using ANI with a threshold ≥95% ^a^. Values and corresponding phenotypes (R: resistant; S: susceptible) of ampicillin (AMP ^b^) and amoxicillin-clavulanic acid (AMC ^c^) are displayed as MICs in mg/L from Etest analyses. Genotypes of interest in the *bla_OXA61_* promoting sequence are shown using their corresponding positions ^d^ (wt: wild type). Genomes in fasta format are available within the ENA database using listed accession numbers ^e^. Mutations and genes expression associated to ciprofloxacin (CIP), erythromycin (ERY), tetracycline (TET) and gentamicin (GEN) resistances are also indicated ^f^. Bold values correspond to resistant isolates.

### Whole genome sequencing and genomes analyses

Each clinical isolate was grown on Columbia blood agar (CBA) plate with 5% sheep’s blood (Thermo Fisher Scientific, MA) and incubated at 37°C in a jar. An Anoxomat microprocessor (Mart Microbiology BV, Lichtenvoorde, The Netherlands) created an microaerobic atmosphere of 79.7% N_2_, 7.1% CO_2_, and 7.1% H_2_ and 6% O_2_. DNA was then extracted from pure bacterial colonies using the MagNA Pure 6 DNA and viral NA SV kit, and DNA purification was performed by bacterial lysis on a MagNA Pure 96 system (Roche Applied Science, Manheim, Germany). Whole genome sequencing (WGS) was performed either on Illumina ISeq 100 (locally at the NRCCH) or Nova Seq 6000 sequencer (Integragen, Evry, France). Sequencing data was analyzed using an in-house pipeline. Specifically, raw reads were cleaned using Sickle v1.33 (Joshi and Fass, 2011) and genomes were assembled using SPAdes v3.15.5 (Bankevich et al., 2012). Generated contigs were filtered depending on their depth (minimum five) and length (minimum 200). Bacterial species were confirmed from *in vitro* Average Nucleotide Identity method using FastANI v1.1 (Jain et al., 2018) and potential sources of contamination were identified using STRUCTURE tool v2.3.4 (Pritchard et al., 2000) combined with host-segregating genomic markers for *C. jejuni* (Thépault et al., 2017) and *C. coli* (Jehanne et al., 2020). Finally, resistance markers (genes and mutations) were isolated from each assembled genomes using Nucleotide-Nucleotide/Protein–Protein BLAST 2.12.0+ (Altschul et al., 1997) command line tool combined with multiple databases: ncbi, card, resfinder, plasmidfinder and an in-house database for *Campylobacter* sp. based on various previous publications (Griggs et al., 2005; Ladely et al., 2009; Qin et al., 2014; Zeng et al., 2014; Fabre et al., 2018; Hormeño et al., 2020; Jehanne et al., 2021).

### Sample preparation for LC–MS/MS analysis

Primary cultures of *C. coli* and *C. jejuni* strains were performed in triplicates on Columbia agar +5% sheep blood plates (bioMérieux, Marcy L’étoile, France) at 37°C for 48 h in microaerobic atmosphere. Sub-cultures were performed under the same conditions and bacterial suspensions were prepared in LC–MS grade water to reach a minimum density of four McFarland. Two hundred microliters of bacterial suspension were transferred into 1.5 mL tubes containing approximately 70 mg of 150–212 μm glass beads (Sigma-Aldrich) and 50 μL of 1 mg/ml recombinant trypsin (Roche) in 150 mM NH_4_HCO_3_ (Sigma-Aldrich) were added to each tube. Bacterial lysis and protein digestion were performed in a thermostated (50°C) Bioruptor ultrasonicator (Diagenode, Lièges, Belgium) for 10 min with ultrasounds being applied for 30 s every minute. Trypsin digestion was stopped by adding 5 μL of formic acid (Sigma-Aldrich). Tubes were then centrifuged at 9600 × *g* for 5 min and 100 μL of supernatant were transferred to a final 2 mL glass vial for LC–MS/MS analysis.

### LC–MS/MS analysis

Samples were analyzed with an Agilent 1290 Infinity liquid chromatography coupled to a SCIEX QTRAP6500+ Triple-quadrupole mass spectrometer equipped with an ESI Turbo V ion source. Ten microliters of samples were injected on the system. Mobile phases were H_2_O + 0.1% formic acid (Buffer A) and ACN + 0.1% formic acid (Buffer B). LC separation was carried out on a Waters Xbridge Peptide BEH C18 (1 mm × 100 mm, particle size 3.5 μm) column heated at 60°C. The analytical gradient was set as follow: 2 to 10% B from 0 to 0.1 min, 10 to 30.5% B from 0.1 to 13.1 min, and 30.5 to 50% B from 13.1 to 15.95 min. Flow rate was set at 100 μL/min. The mass spectrometer ion source temperature was 550°C and the ion spray voltage set at 5500 V. Curtain gas, nebulizer gas (GS1) and heating gas (GS2) were, respectively, set at 50, 70, and 60 psi.

### MRM assay development

The amino-acid sequence of Bla_OXA61_ beta-lactamase was digested *in silico* with trypsin using Skyline software (version 22.2.0.351) to generate every potential surrogate peptide. An initial MRM method was built monitoring three y ion-type fragments for each doubly and triply charged putative peptides to identify the ones detectable in strains known to express high amounts of Bla_OXA61_. All peptides selected during the first screening step were included in a second MRM method to monitor every y and b ions of the most intense charge state. Signal specificity was confirmed when at least eight transitions were detected at the same retention time in strains known to express *bla_OXA61_* and when no signal was observed for strains known to have intrinsic repression of the beta-lactamase expression. A final MRM assay was built with the three most intense fragment ions of each Bla_OXA61_ peptide as well as three y ions for five peptides derived from *Campylobacter* ribosomal proteins (Supplementary Table 1).

### Peak detection and relative quantification

Raw chromatograms were analyzed using Skyline software. For each peptide, peak integration was manually checked and curated when necessary to ensure correct quantification. Label-free quantification strategies have now been widely applied and demonstrated their robustness for quantification of proteins in biological samples (Cecchini et al., 2018; Fournier et al., 2021; Pivard et al., 2023). Those approaches rely on the assumption that signal of the most intense transitions of the best flying peptide correlate with protein abundance and can therefore be used as an indicator of protein amounts (Ludwig et al., 2012). Here, the area of the three transitions of three Bla_OXA61_ peptides (EQAILLFR, YLDELVK and IDTFWLDNSLK) were summed to compensate for potential slight variations in fragmentation and trypsin digestion repeatability across runs. Moreover, to take into account bacterial load variations across samples, the sum of Bla_OXA61_ peptides is expressed relative to signals of five housekeeping peptides derived from ribosomal proteins used as indicators of bacterial density due to their quantotypic properties (Cecchini et al., 2018; Pivard et al., 2023). In other words, quantification values are obtained using Equation 1:


(1) $$Quantification=\frac{\sum \;of\;the\;3\;transitions\;of3\;BlaOXA61\;peptides}{\sum \;of\;the\;3\;transitions\;of5\;ribosomal\;peptides}$$

## Results

### Genomic analyses

**Figure 1:**
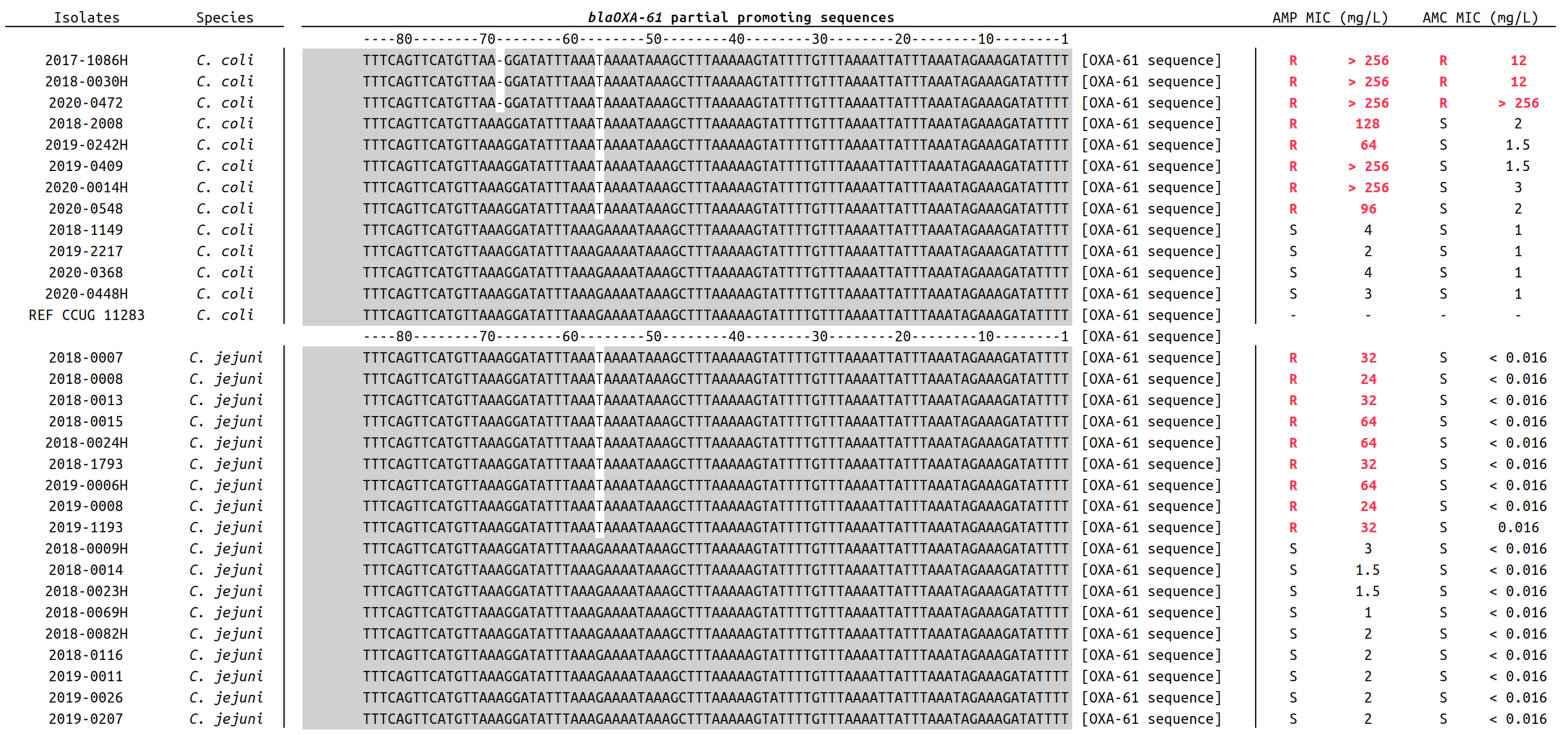
Alignment of *bla_OXA61_* promoting sequences in 18 *C. jejuni* and 12 *C. coli* included in the present study. For each isolate, ampicillin and amoxicillin-clavulanic acid MICs are indicated. Promoting regions were extracted from every assembled genome using Nucleotide-Nucleotide BLAST 2.12.0+ (Altschul et al., 1997), and sequences were aligned using Muscle v3.8.1551 (Edgar, 2004). Relevant genotypes are here highlighted in white at positions −57 and −69.

*C. jejuni* and *C. coli* genomes were properly assembled and species were confirmed with average scores of 97.57% (±0.14) and 98.79% (±0.13) for *C. jejuni* and *C. coli* isolates, respectively (Table 1). The average genomes size was 1.72 Mbp (±67 kbp) with an average contigs number of 80 (±196; isolate fasta “2018–0015” is comprised of more than 1000 contigs which significantly increases the data) and an average contig length of 42 kbp (±15.8 kbp). Moreover, genomes GC % was about 32% (±2.2%) and the average number of coding sequences (CDS) was 1,766 (±66), in accordance with previously published data (Pearson et al., 2013; He et al., 2020). Additionally, most of the selected clinical isolates were attributed to the chicken reservoir using source attribution models (*data not shown*), which represents 63.3% of the dataset. Genomic antimicrobial resistance (AMR) analysis allowed to display the G57T mutation in the *bla_OXA61_* promoting region among every AMP-R *C. jejuni* and *C. coli* isolates, highlighting the importance of this marker in ampicillin resistance for Campylobacters (Zeng et al., 2014). A supplemental deletion in position A69 of that promoting sequence was also exclusively identified among all three AMC-R *C. coli* isolates (two from chicken and one from pig according to the source attribution markers), as shown in Figure 1. The use of mass-spectrometry was used to estimate the impact of such deletion on the expression of Bla_OXA61_.

### MRM analyses

To quantitatively assess the expression levels of Bla_OXA61_, each strain was grown in triplicates and trypsin-digested bacterial lysates were analyzed by targeted mass spectrometry for the detection of Bla_OXA61_. Due to variations observed in the relative intensities of ribosomal peptides (used as housekeeping standards for cell number and expression level normalization, as described in material and methods) between *C. jejuni* and *C. coli*, Bla_OXA61_ expression levels were only compared within the same species.

**Figure 2:**
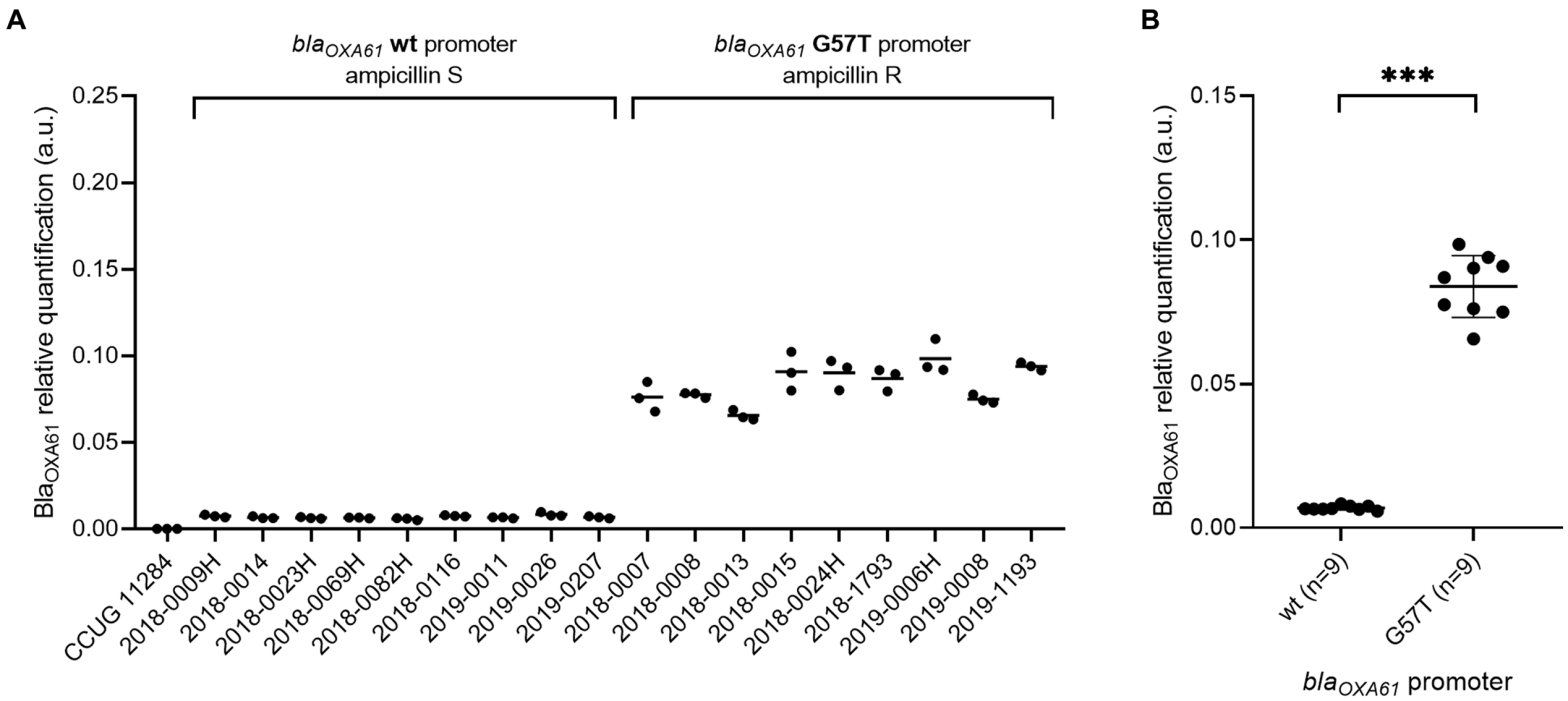
Bla_OXA61_ relative quantification in ampicillin-susceptible and resistant *C. jejuni* isolates. **(A)** Each strain individually. **(B)** Strains grouped according to the *bla_OXA61_*-promoting sequences. Data are expressed in arbitrary units. In panel **(B)**, the mean of triplicates was used to perform a Mann–Whitney *U* test. ***p value <0.001. wt, wild type; S, susceptible; R, resistant. No signal was observed for *C. jejuni* reference CCUG 11284.

**Figure 3:**
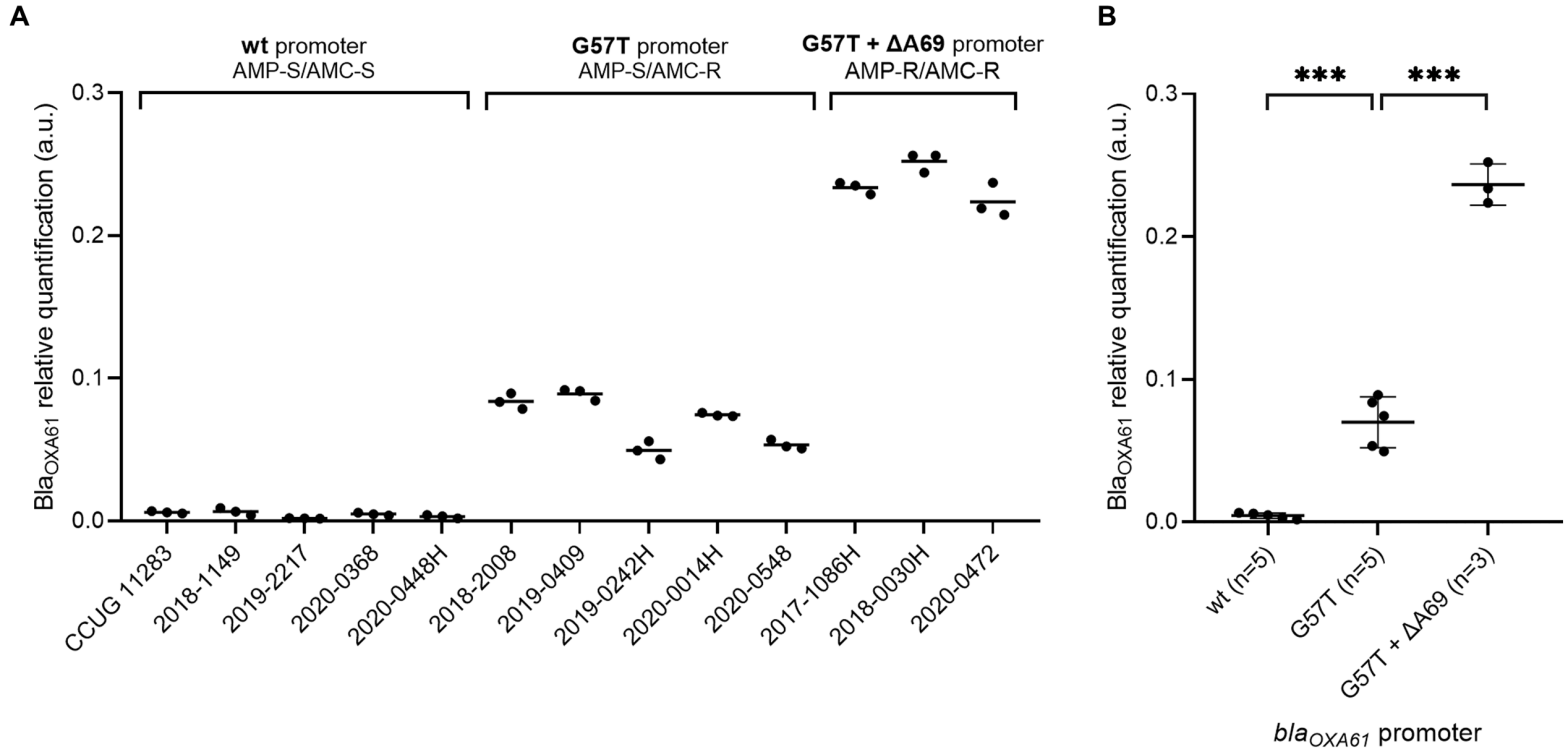
Expression levels of Bla_OXA61_ in *C. coli* isolates with wild type, G57T or G57T + ∆A69 promoters. **(A)** Each strain individually. **(B)** Strains grouped according to the *bla_OXA61_*-promoting sequences. Data are expressed in arbitrary units. In panel **(B)**, the mean of triplicates was used to perform one-way ANOVA. ***p-value <0.001. wt, wild type; AMP, ampicillin; AMC, amoxicillin + clavulanic acid; S, susceptible; R, resistant.

In comparison to the wild type promoter, the addition of the G57T mutation in *bla_OXA61_* promoting region leads to an increase of the beta-lactamase expression and confers resistance to ampicillin for both *Campylobacter* species (Figures 2A, 3A). Relative quantification revealed a significant 15-fold and 16-fold increase in overexpression of Bla_OXA61_ compared to the ampicillin susceptible (wild type promoter) in *C. jejuni* and *C. coli* isolates, respectively (Figures 2B, 3B).

The combination of the G57T mutation with a supplemental deletion in position A69 was associated with a significant overexpression of Bla_OXA61_ and resistance to amoxicillin-clavulanic acid in the three *C. coli* strains included in the present study. Indeed, a significant 54 and 16-fold increase was observed compared with strains harboring a WT or a G57T *bla_OXA61_* promoter (Figure 3B), respectively.

## Data availability

Corresponding genome accession numbers of each clinical isolates are available in Table 1 and under ENA Study number PRJEB63218. MRM raw data and transition list are available via PASSEL with the accession number PASS05834.^3^

## Discussion

*Campylobacter* resistance to antimicrobials is concerning (Wieczorek and Osek, 2013). However, resistance to amoxicillin combined to clavulanic acid beta lactamase-inhibitor among human *Campylobacter* isolates remains sparse (Deckert et al., 2013; Post et al., 2017; Wallace et al., 2021), even though ampicillin resistance rates in poultry reservoirs are high (Casagrande Proietti et al., 2020; Béjaoui et al., 2022; Gharbi et al., 2023) (up to 73%). This current study proposes a combined genomic and proteomic approach to characterize the mechanism responsible for amoxicillin-clavulanic acid (AMC) resistance in *Campylobacter* clinical isolates, specifically *C. coli*. This experimental strategy used both molecular and mass spectrometry analyzes and highlighted a deletion at position A69 in the *bla_OXA61_* promoting region contributing to a significant increase of the beta-lactamase expression, which is associated with amoxicillin-clavulanic acid resistance.

The existence of beta-lactamases among *C. jejuni* and *C. coli* isolates is now well described, such as *bla_OXA61_*, *bla_OXA489_* or *bla_OXA193_*, with *bla_OXA61_* being the most frequently observed (Cobo-Díaz et al., 2021). Even though their expression has been widely associated with aminopenicillin resistance, many previous studies are based uniquely on the presence or absence of beta-lactamase genes without taking into account the importance of the promoting region (Gharbi et al., 2023). As a matter of fact, a single nucleotide at position 57 (G → T) in this specific sequence is associated with ampicillin resistance (Zeng et al., 2014). Activity of beta-lactamase inhibitors such as clavulanic acid used in combination to amoxicillin may be significantly impaired in case of *bla_OXA61_* overexpression, as shown in a previous study using PCR and qPCR amplification (Casagrande Proietti et al., 2020).

In the present study, LC–MS/MS allowed to confirm that the G57T mutation in the *bla_OXA61_* promoting region is responsible for an increased expression of the beta-lactamase, thus conferring ampicillin resistance. However, every *C. jejuni* and *C. coli* clinical isolates which only displayed that single mutation remain highly susceptible to the amoxicillin-clavulanic acid activity. Nevertheless, we showed that three AMC-resistant *C. coli* isolates displayed a supplemental A69 deletion responsible for an overexpression of Bla_OXA61_. Those high levels of Bla_OXA61_ production seems to fully encounter clavulanic acid inhibitor activity. Additional *C. coli* isolates with both G57T + ∆A69 genotypes but also isolates showing ∆A69 only would need to be collected to ensure more robust statistical analysis and therefore better assess the correlation between their resistance phenotype and the expression level of Bla_OXA61_.

According to Tajada et al. (1996) the great activity of clavulanic acid in *C. jejuni* and *C. coli* is due to its ability to bind Penicillin Binding Proteins with lower MICs in *C. jejuni* compared to *C. coli*. It is therefore maybe not surprising to observe amoxicillin-clavulanic acid resistance only in *C. coli*. Amoxicillin-clavulanic acid resistance remains however very rare among *C. jejuni* and *C. coli* clinical isolates. Nevertheless, such broadened resistance spectrum conferred by overexpression of a beta-lactamases due to point mutations in the promoter region is not unusual, as it has been well described in other species especially *E. coli* (Corvec et al., 2002; Tracz et al., 2005; Singh et al., 2019). Site-directed mutagenesis to induce changes in the *bla_OXA61_* promoting sequence of amoxicillin-clavulanic acid and ampicillin susceptible isolates could however be performed in order to fully observe the impact on the gene expression.

In conclusion, combined genomic-proteomic approach here allowed us to identify a new *bla_OXA61_* promoting region displaying both G57T mutation and an uncharacterized A69 deletion which leads to a significative overexpression of this beta-lactamase and may be responsible for amoxicillin-clavulanic acid resistance. These results are even more valuable as we lack consensus data on such phenotypes. Indeed, *C. jejuni* and *C. coli* breakpoints for amoxicillin-clavulanic acid today are based on that for other species, such as the use of EUCAST *Enterobacteriaceae* recommendations. Although this mechanism is rare among *C. coli* clinical isolates, it needs to be seriously considered since *C. coli* species isolates can easily adapt to their environment.

## Data availability statement

The datasets presented in this study can be found in online repositories. The names of the repository/repositories and accession number(s) can be found in the article/Supplementary material.

## Ethics statement

Ethical review and approval was not required for the study in accordance with the local legislation and institutional requirements. Written informed consent for participation was not required for this study in accordance with the national legislation and the institutional requirements. Written informed consent was not obtained from the individual(s) for the publication of any potentially identifiable images or data included in this article.

## Author contributions

FD: Formal analysis, Methodology, Validation, Writing – original draft, Writing – review & editing. QJ: Formal analysis, Writing review & editing, Software. LB: Methodology, Writing – review & editing. JA: Methodology, Writing – review & editing. RP: Methodology, Writing – review & editing. CD: Methodology, Writing – review & editing. AD: Methodology, Writing – review & editing. MJ: Writing – review & editing. RB: Writing – review & editing, Methodology. FV: Writing – review & editing. JL: Writing – review & editing. PL: Writing – review & editing, Data curation, Formal analysis, Funding acquisition, Methodology, Project administration, Supervision, Validation, Writing – original draft.

## Footnotes

^1^http://www.eucast.org

^2^https://www.sfm-microbiologie.org/boutique/comite-de-lantibiograme-de-la-sfm-casfm/

^3^https://db.systemsbiology.net/sbeams/cgi/PeptideAtlas/PASS_View
