# Supplementary material for: Combined genomic-proteomic approach in the identification of Campylobacter coli amoxicillin-clavulanic acid resistance mechanism in clinical isolates
Source: Front Microbiol. 2023 Nov 9;14:1285236. doi: 10.3389/fmicb.2023.1285236 (PMC10666280; doi:10.3389/fmicb.2023.1285236)
Supplement: Supplementary file 1 [file Table_1.docx]

| Protein | Peptide sequence | Precursor charge state | Fragment ions | Used for |
| --- | --- | --- | --- | --- |
| BlaOXA61 | ILNNWFK | 2+ | y6, y5, b2 | Validation |
|  | TWASNDFSR | 2+ | y7, y6, b2 | Validation |
|  | AMETFSPASTFK | 2+ | y9, y8, y7 | Validation |
|  | IFNALIALDSGVIK | 2+ | y9, y8, b4 | Validation |
|  | YSNVLAFK | 2+ | y7, y6, y3 | Validation |
|  | TMQEYLNK | 2+ | y6, y5, y4 | Validation |
|  | IDTFWLDNSLK | 2+ | y7, y6, b3 | Quantification |
|  | EQAILLFR | 2+ | y6, y4, y3 | Quantification |
|  | EMIYLK | 2+ | y5, y4, y3 | Validation |
|  | NMENLELFGK | 2+ | y8, y7, y2 | Validation |
|  | TGFNDEQK | 2+ | y7, y6, y5 | Validation |
|  | YLDELVK | 2+ | y6, y5, b2 | Quantification |
| 50S ribosomal protein L25 | LDVGDALLVR | 2+ | y7, y6, y5 | Quantification |
| 30S ribosomal protein S5 | SLGSNNSANVVR | 2+ | y10, y6, y2 | Quantification |
| 50S ribosomal protein L13 | HSGYFGSVK | 2+ | y8, y7, y6 | Quantification |
| 30S ribosomal protein S4 | FMYGVSEK | 2+ | y7, y6, y5 | Quantification |
| 30S ribosomal protein S7 | LAAELLDAANSK | 2+ | y10, y7, y6 | Quantification |

**Supplemental Table 1. Peptides included in the MRM assay.**
